# Prevalence of Hypoproteinemia and Hypoalbuminemia in Pregnant Women from Three Different Socioeconomic Populations

**DOI:** 10.3390/ijerph17176275

**Published:** 2020-08-28

**Authors:** Sagrario Gómez-Cantarino, M. Teresa Agulló-Ortuño, Mercedes de Dios-Aguado, M. Idoia Ugarte-Gurrutxaga, Carmen Bouzas-Mosquera

**Affiliations:** 1 Department of Nursing, Physiotherapy and Occupational Therapy, Faculty of Physiotherapy and Nursing, Universidad de Castilla-La Mancha (UCLM), 45071 Toledo, Spain; Sagrario.Gomez@uclm.es (S.G.-C.); Maria.Ugarte@uclm.es (M.I.U.-G.); Carmen.Bouzas@uclm.es (C.B.-M.); 2 Primary Health Care Center, Servicio de Salud de Castilla-La Mancha (SESCAM), 45313 Toledo, Spain; mded@sescam.jccm.es

**Keywords:** pregnancy, plasma proteins, primary care, women’s health

## Abstract

Protein requirements of pregnant women are increased due to anatomical and physiological changes. However, optimal levels of plasma proteins do not receive adequate attention from health professionals and researchers. We aimed to evaluate the plasma protein status in pregnant women receiving care at health centers, with the intention of identifying potential deficiency states and their relationship with quality of life during pregnancy. This is a population-based, prospective, and observational study among a cohort of 215 pregnant women from three different socioeconomic areas (urban, semi-urban, and rural). Blood samples in the first (T1), second (T2), and third (T3) trimester of pregnancy were obtained to quantify the proteins and albumin levels. Statically significant differences regarding the age of pregnant women (*p* = 0.002), education status (*p* = 0.034), and socioeconomic level (*p* = 0.000), were found among groups. Prevalence of protein and albumin deficits was much higher in women from rural and semi-urban areas than in women from urban areas (*p* = 0.001). Moreover, these deficits were associated with the appearance of edema. Plasma total protein deficit could be an undervalued public health problem in pregnant women receiving prenatal care that could affect the quality of life in the gestational period. It would be important to establish reference intervals for plasma protein monitoring in each trimester of pregnancy, and protein levels should be measured routinely throughout pregnancy.

## 1. Introduction

The nutritional status of women before and during pregnancy is considered a determinant factor in maternal and neonatal health outcomes [1,2]. Because pregnant women experience significant anatomical and physiological changes [3], they require an increased intake of protein as protein deposition in maternal organs and fetal tissues increases throughout pregnancy, mainly in the third trimester [4]. It has been described that low- and higher-protein intakes during pregnancy are associated with detrimental effects on both the pregnant women and the developing fetus [5,6]. However, there are some controversies regarding this assertion, mainly due to considerable variations in protein intake during pregnancy, and different nutritional patterns in the population studied [7].

From a biochemical viewpoint, nutritional status can be determined through the measurement of blood parameters, such as hemoglobin level, circulating iron, vitamins, and trace element concentrations [8,9]. The urine protein level of pregnant women has aroused the interest of researchers because of the strong relationship between proteinuria and the onset of preeclampsia or other syndromes [10]. However, the study of low protein levels in blood has not received the same interest, except in malnourished populations of low-and middle-income countries [11]. On the other hand, the concentration of blood biomarkers in pregnant women is influenced by plasma volume expansion [12,13], and causes of hypoproteinemia in pregnancies can be very varied, including hemodilution, increased kidney clearance, and higher usage of proteins on behalf of the fetus and maternal organs.

Albumin comprises 50–60% of the total protein content of blood. Hypoalbuminemia usually happens in combination with hypoproteinemia or dietary protein restrictions, and it is associated with poor quality of live [14]. The pathogenesis of hypoalbuminemia is still unclear, although malnutrition, enteropathies, inflammatory status, heart or kidney damage, and pregnancy are recognized causes.

Despite the importance of maintaining an adequate plasma protein level in pregnant women to avoid undesirable effects on both the fetus and the mother, there are not enough studies on the prevalence of this deficit, and data from descriptive and analytical assessment remain insufficient. Therefore, the aim of the present work is to evaluate the plasma protein status in pregnant women receiving care at public health centers of three different socioeconomic areas (urban, semi-urban, and rural), and its relationship with maternal and fetal outcomes.

## 2. Materials and Methods

### 2.1. Study Design

This is a population-based, prospective, and observational study in a cohort of pregnant women from three different geographical areas of the Autonomous Community of Castilla-La Mancha (Spain). Eligible participants were followed throughout pregnancy at four health centers: Buenavista Health Centre and Santa Bárbara Health Centre in Toledo (urban area); Polán Health Center (semi-urban area); and Yepes Health Center (rural area).

We included a total of 215 women over 18 years old with singleton pregnancy, who were followed throughout pregnancy at health centers. Women with pre-gestational pathologies, such as chronic hypertension, diabetes, obesity, preeclampsia in previous pregnancies, and presence of kidney or autoimmune diseases, were excluded. Gestational age was evaluated from ultrasound scans performed at the first visit to the health center. During the first and subsequent visits, all women provided answers to anamnesis questionnaire about obstetric history, prenatal care, socioeconomic, and anthropometric data. Information regarding pregnancy outcomes was obtained from medical records. Data collection was performed between January 2016 and December 2017.

### 2.2. Total Protein and Albumin Level Evaluation

Blood samples were collected by brachial venipuncture into lithium heparin-tubes, centrifuged, and transported to a central laboratory for analysis. Metabolites were quantified by standard chemical and enzymatic commercial methods in an ADVIA^®^2400 Automated Clinical Chemistry System (Bayer Healthcare LLC, Whippany, NJ, USA). Reference intervals are 6.4–8.3 g/dL for plasma total proteins, and 3.4–4.8 g/dL for plasma albumin. According to the protocol stablished in our Public Health System, total protein and albumin levels were determined in the first trimester (T1; between week 6 and 9 of gestation), second trimester (T2; week 26 of gestation), and third trimester (T3; week 35 of gestation).

### 2.3. Statistical Analysis

Analyses were performed using the SPSS/PC ver. 25.0 statistical package (IBM, Chicago, IL, USA). Median, mean, and standard deviation (SD) or 95% confidence intervals were used to describe the analyzed parameters. Differences in distributions of individual parameters between study subgroups were analyzed using ANOVA or Kruskal–Wallis tests if the distribution showed a significant deviation from normality. A Chi-Square test was used for categorical data. Relations among variables were assessed using Pearson or Spearman correlation coefficients. All the tests used were two-tailed, and *p* < 0.05 was considered significant.

### 2.4. Ethical Consideration

The study has been carried out to the highest ethical standards, in accordance with the Declaration of Helsinki, International Conference on Harmonization Guidelines for Good Clinical Practice, and local ethical and legal requirements. All women signed written informed consent for inclusion before participating in the study. The Research and Clinical Ethics Committee of “Complejo Hospitalario de Toledo” approved the study objective and procedures (2015/125).

## 3. Results

### 3.1. Socio-Demographic Characteristics

Table 1 shows the basic characteristics of the participants in this study. There were statistically significant differences between the age of pregnant women of urban areas and rural residents (*p* = 0.004), but no differences were found between the age of urban and semi-urban women (*p* = 0.261), and between the age of semi-urban and rural women (*p* = 0.206).

Based on educational status, we found significant differences among the groups studied (*p* = 0.034), with urban and semi-urban groups having a higher percentage of women with higher education levels. As expected, we found statistically significant differences (*p* = 0.000) in the socioeconomic level of women, with most rural women from a lower economic group (65.9%). There was also a higher percentage of housewives in the rural area (*p* = 0.000).

### 3.2. Anthropometric Characteristics and Obstetric Data

In our study, only 3.72% of participants were underweight (body mass index (BMI) < 18.5 kg/m^2^), but 33.02% of women were overweight (BMI > 25 kg/m^2^) at the beginning of the pregnancy. We only found statistically significant differences in the BMI at T2 between urban women and women from semi-urban or rural areas (*p* = 0.026), with BMI of the women from the urban area lower than that of women from semi-urban and rural areas. The weight gain was higher in women from semi-urban areas and very similar between women of urban and rural areas (Table 1).

There were no statistically significant differences between the three groups studied regarding the mean birth weight of the newborns. Only 12 newborns had a low birth weight (<2500 g: 8 of them were children from mothers from the rural area (6.2%); 3 were children from mothers from the semi-rural area (5.77%), and 1 was from a mother from the urban area (2.94%)). Likewise, there were no differences between the three groups regarding the Apgar score [15] of the newborns.

### 3.3. Prevalence of Deficit of Plasma Total Protein and Albumin

**Figure 1:**
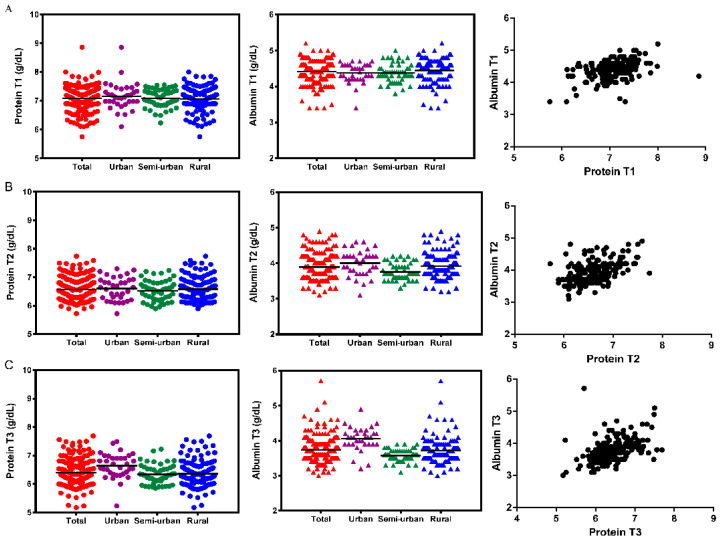
Levels of plasma total protein and albumin among pregnant women at T1 (**A**), T2 (**B**), and T3 (**C**) trimesters of pregnancy. Left panel: plasma total protein level; middle panel: plasma albumin level; right panel: correlation between plasma total protein and albumin levels in the total population (T1: Spearman’s Rho, ρ = 0.457, *p* = 0.000; T2: ρ = 0.543, *p* = 0.000; T3: ρ = 0.539, *p* = 0.000).

The plasma concentration of total proteins decreased progressively over the three trimesters of pregnancy in our cohort, with statistically significant differences (*p* = 0.000). Nevertheless, we should point out that the mean values of the plasma protein concentration in the subgroup of women from urban area were higher than those of the subgroups of women from the semi-urban and rural areas, mainly in the third trimester of pregnancy with statistically significant differences (Table 2 and Figure 1). Thus, we found statistically significant differences (*p* = 0.011) in the mean decrease in the protein level during T3 between women from urban areas (−0.665 ± 0.638 g/dL) and women from semi-urban (−0.769 ± 0.317 g/dL) or rural areas (−0.830 ± 0.395 g/dL).

Similarly, the plasma concentration of albumin decreased progressively over the three trimesters of pregnancy (*p* = 0.000), (Table 2 and Figure 1). Again, the mean levels of plasma albumin during the second and third trimesters were higher in women from the urban area. We found statistically significant differences in T2 between women from the urban area and women from the semi-urban area (*p* = 0.001), and between women from semi-urban and rural areas (*p* = 0.005); and in T3 between women from the urban area and women from semi-urban (*p* = 0.000) or rural areas (*p* = 0.000). We also found differences in the plasma albumin level between women from semi-urban areas and women from rural areas at T2 (*p* = 0.005) and T3 (*p* = 0.038) trimesters of pregnancy, but curiously the albumin level in women from rural areas was slightly higher.

Once again, we found statistically significant differences among the three groups of women regarding the quantification of albumin decrease during the pregnancy, both in T2 and T3 trimesters. There was a difference between the albumin level mean decrease in women from urban areas (−0.368 ± 0.388 g/dL) and women from semi-urbans (−0.621 ± 0.275 g/dL), at T2 (*p* = 0.004). There were also differences between women from urban areas (−0.315 ± 0.321 g/dL) and women from semi-urban (−0.802 ± 0.268 g/dL) or from rural areas (−0.724 ± 0.481 g/dL), at T3 (*p* = 0.000 in both cases). The correlation between plasma total protein and plasma albumin is shown in Figure 1 (right panel).

As shown in Table 3, the prevalence of a deficit or a low plasma total protein level (<6.3 g/dL) increased progressively throughout pregnancy. However, the prevalence of protein deficit was higher in women from rural and semi-urban areas than in women from urban areas, mainly during the third trimester of pregnancy (*p* = 0.001).

There was no plasma albumin deficit (<3.4 g/dL) in T1 of the pregnancy in any of groups from our cohort. However, there was a slight prevalence of hypoalbuminemia in the second trimester of pregnancy, which increased during the third trimester. Again, women from semi-urban and rural areas were the most affected with respect to this deficit (Table 3). On the other hand, we found a statistical relation between primigravidae women and a low level of total protein in T3 compared to multiparous women (6.317 ± 0.436 vs. 6.467 ± 0.431 g/dL; *p* = 0.013).

### 3.4. Relationship between Complications during Pregnancy and Plasma Total Protein and Albumin Levels

Table 4 shows the association between plasma total protein and albumin levels with the appearance of edema or swelling in women throughout pregnancy. We found statistically significant differences in T2 and T3 regarding the level of total protein (*p* = 0.036 and *p* = 0.008), and level of albumin in plasma (*p* = 0.007 and *p* = 0.001) between women with edema and those without. The appearance of edemas was also more frequent in women who lived in semi-urban and rural areas than in urban women, with significant differences in T2 (*p* = 0.000) and T3 (*p* = 0.000) (Table 1). We found no relationship between the appearance of edema and other maternal factors, such as age, parity, BMI, weight gain, and socioeconomic or education level.

We also did not find a relationship between the plasma total protein or albumin levels with other symptoms of pregnancy complications, such as muscle cramps or weakness and fatigue manifested by women included in this study.

In our cohort, only 12 newborns had low birth weight, and in these cases, we found no association with total protein or albumin levels in mothers’ plasma in any of the trimesters of pregnancy. However, mothers with babies with low birth weight had lower levels of total protein at T1 (6.889 ± 0.520 g/dL) than mothers with normal birth weight babies (7.103 ± 0.375 g/dL), although without statistical significance (*p* = 0.064; Mann–Whitney U test). The correlation between the maternal protein level and newborn weight was as follows: T1: ρ = −0.064, *p* = 0.348; T2: ρ = −0.088, *p* = 0.198; T3: ρ = −0.069, *p* = 0.315. Similarly, albumin levels were slightly lower in mothers with babies underweight at birth, although without statistical significance. The correlation between the maternal albumin level and newborn weight was as follows: T1: ρ = −0.037, *p* = 0.593; T2: ρ = −0.033, *p* = 0.632; T3: ρ = −0.079, *p* = 0.252.

We also found no association between maternal levels of protein or albumin and the Apgar score of newborns. The correlation between the maternal protein level and Apgar score was as follows: T1: ρ = −0.109, *p* = 0.111; T2: ρ = −0.081, *p* = 0.239; T3: ρ = −0.058, *p* = 0.401; and between the maternal albumin level and Apgar score: T1: ρ = −0.074, *p* = 0.282; T2: ρ = −0.089, *p* = 0.195; T3: ρ = −0.101, *p* = 0.143.

## 4. Discussion

In this work, we studied a cohort of pregnant women from three different socioeconomic areas in our country. Although advanced maternal age has been associated with increased adverse pregnancy outcomes, it is becoming increasingly common in Western societies [16,17]. Among reasons to delay pregnancy are cultural and value shifts, a higher educational level reached by women, economic uncertainty, or unstable labor markets, etc. [18]. According to these observations, we found significant differences among women from the three socioeconomic areas studied. Urban dwellers have a higher education and economic level, greater incorporation into the world of work, and a more advanced maternal age.

The pregnant woman undergoes anatomical and physiological changes to provide the right conditions for pregnancy success, which implies a gain in weight. Nevertheless, paying special attention to diet and exercise during pregnancy can reduce certain health risks [19]. In our cohort, women from urban areas had a lower BMI through the pregnancy. The weight gain was higher in women from semi-urban areas and lower in women from rural areas, a fact that was not related to adverse pregnancy outcomes. In our opinion, this could be due to different maternal dietary habits and socioeconomic conditions of the three groups of women. Other authors have associated pre-pregnancy BMI and weight gain during pregnancy with the offspring’s birth weight [11], but this association was not found in our study.

Most of the studies carried out so far are focused on the nutritional point of view, studying the relationship between the protein diet followed during pregnancy and its effects of the mother and the fetus. However, the consequences of low levels of maternal plasma total proteins have been neglected.

Our results show a high prevalence of hypoproteinemia in the second and third trimesters of pregnancy, mainly in woman from semi-urban and rural areas. The albumin deficit is like that of protein, although in a smaller proportion. This could indicate that, although albumin is the most abundant protein in the blood, other proteins could play an important role in this blood protein deficit in pregnancy. Further studies are necessary to clarify this. In addition, these results could be explained by an increase in the maternal–fetal transfer of nutrients in the later stages of pregnancy because of faster fetal growth [20]. Moreover, measurement of blood proteins is influenced by plasma volume expansion, and comparisons between pregnant women can be difficult due to the wide individual variation, the measurement method employed, or the reference group used [12,13].

In this study, both plasma protein and albumin deficiencies were associated with the appearance of edema in T2 and T3. Although swelling and edema are very common during pregnancy, sometimes they can be signals of more serious underlying problems. Professional recommendations in these cases are to eat a healthy diet, perform moderately intense exercise, wear comfortable clothes, and other options designed to reduce venous pressure. Unfortunately, few biochemical studies have been conducted on the source of this discomfort, even more so when plasma protein deficit is one of the identified causes of these symptoms, and the single recommendations do not take into account the changes needed during different stages of pregnancy [21,22,23]. Thus, the prevalence of hypoproteinemia assessed in this work could be an undervalued public health problem in pregnant women receiving prenatal care.

On the other hand, our public health system has no reference intervals for plasma total proteins and albumin in pregnant women, and we used reference intervals in the healthy population for this study. This is an important limitation because the prevalence of protein deficiency could be overestimated, and these results need to be taken as an indication. Our study had an unequal number of participants in the three socioeconomic areas. This is because the number of pregnant women was different in the areas studied during the period of patient inclusion. Another limitation is that no quantitative or qualitative evaluation was performed on the protein dietary consumption of women included in the study. However, a fact that we would like to point out is that our country is among the top 10 Healthiest Countries in the World, with the highest life expectancy at birth among European Union nations, and a public primary care of high quality and healthy eating habits based on the Mediterranean diet are among the main arguments for this qualification [24,25]. The strength of our study lies in the fact that our results can serve to alert health professionals to the development and promotion of better prevention strategies in pregnant women.

## 5. Conclusions

The association between plasma proteins level in pregnancy and unwanted effects is complex and is not well understand. This study has shown a high prevalence of hypoproteinemia and hypoalbuminemia, and this happens in pregnant women from three different socioeconomic strata who received prenatal care in public health centers. This prevalence was more noticeable in women from semi-urban and rural areas. Those differences may be due to the different nutritional patterns, purchasing power, and education level among women. The main consequence of plasma protein and albumin deficiencies identified in our study is the appearance of edema in pregnant women during the second and third trimesters of pregnancy, with the discomfort and possible side effects that it produces. Fortunately, we have not found consequences of hypoproteinemia in the offspring, although we have observed a trend between mothers with a protein deficit and newborns with low weight.

Our results suggest the need to identify specific cut-off points for each trimester of pregnancy in order to establish an accurate diagnosis of plasma protein deficit in pregnant women. Considering that total protein and albumin are biomarkers determined in everyday routine practice, our recommendation is that they should be measured during the pregnancy check-up and management used in healthcare, to avoid unwanted complications.

## Figures and Tables

**Table 1:** Characteristics of 215 women included in the study.

	Total	Urban	Semi-Urban	Rural	*p* *
N	215	34	52	129	
Maternal age (years)	31.78 ± 5.14	34.09 ± 3.16	32.35 ± 4.71	30.94 ± 5.52	0.004 ^1^
Housewife	57 (26.51)	3 (8.82)	5 (9.62)	49 (37.98)	0.000 ^2^
Education level (n, %)				7 (5.42)	0.034
Illiterate	7 (3.26)	0	0	41 (31.78)	
Primary school	59 (27.44)	3 (8.82)	15 (28.85)	60 (46.51)	
Secondary school	108 (50.23)	23 (67.65)	25 (48.07)	21 (16.28)	
Bachelor or higher	41 (19.07)	8 (23.53)	12 (23.08)		
Socioeconomic level					0.000
Low	102 (47.44)	0	17 (32.69)	85 (65.89)	
Medium	74 (34.42)	12 (35.30)	30 (57.69)	32 (24.81)	
High	39 (18.14)	22 (64.70)	5 (9.62)	12 (9.30)	
Primigravidae (n, %)	110 (51.16)	18 (52.94)	25 (48.08)	67 (51.94)	0.873
Newborn weight (kg)	3.32 ± 0.46	3.38 ± 0.45	3.31 ± 0.44	3.31 ± 0.47	0.763
Apgar score (1–10; a.u.) ^3^	8.95 ± 0.36	9.00 ± 0.35	8.92 ± 0.48	8.95 ± 0.30	0.618
BMI (kg/m^2^)					
T1	24.11 ± 4.04	22.89 ± 2.83	24.20 ± 4.64	24.39 ± 4.03	0.118
T2	25.84 ± 4.18	24.17 ± 2.98	26.01 ± 4.34	26.21 ± 4.31	0.026 ^4^
T3	27.72 ± 4.17	26.06 ± 3.08	28.19 ± 4.35	27.97 ± 4.27	0.069
Weight gain (kg)	9.34 ± 4.12	9.09 ± 4.47	10.52 ± 4.52	8.93 ± 3.79	0.012 ^5^
Pregnancy complications (n, %)					
Edema					
T1					
T2	2 (0.93)	0	1 (1.92)	1 (0.78)	0.635
T3	43 (20.00)	0	24 (46.15)	19 (14.73)	0
Cramps (n, %)	58 (26.98)	1 (2.94)	31 (59.62)	26 (20.16)	0
T1					
T2	19 (8.84)	4 (11.76)	3 (5.77)	12 (9.30)	0.109
T3	72 (33.49)	13 (38.24)	14 (26.92)	45 (34.88)	0.26
Weakness (n, %)	86 (40.00)	14 (41.18)	22 (42.31)	50 (38.76)	0.589
T1					
T2	181 (84.19)	30 (88.24)	50 (71.15)	101 (78.58)	0.107
T3	166 (77.21)	25 (73.53)	41 (78.85)	100 (77.52)	0.122
	180 (83.72)	33 (97.06)	43 (86.69)	104 (80.62)	0.166

Mean ± SD or frequency (%) values are shown. * ANOVA (or Kruskal–Wallis test if no normality) or chi-square test performed when appropriate. ^1^ ANOVA test. Differences statistically significant between urban group and rural group (Tuckey test, *p* = 0.004). ^2^ Differences statistically significant between urban and semi-urban groups versus rural group. ^3^ 5-min Apgar score (color, heart rate, reflexes, muscle tone, and respiration). a.u.: arbitrary units. ^4^ Kruskal–Wallis test. Differences statistically significant between urban group and rural group (pairwise comparisons, *p* = 0.026). ^5^ Kruskal–Wallis test. Differences statistically significant between semi-urban group and rural group (pairwise comparisons, *p* = 0.011). BMI: Body Mass Index; T1: first trimester of gestation; T2: second trimester of gestation; T3: third trimester of gestation.

**Table 2:** Plasma total protein and albumin levels in women included in the study.

	Total Protein (g/dL)				Albumin (g/dL)			
	T1	T2	T3	*p* *	T1	T2	T3	*p* *
Total	7.082 ± 0.39	6.569 ± 0.39	6.398 ± 0.44	0.000 ^1^	4.416 ± 0.29	3.895 ± 0.34	3.738 ± 0.36	0.000 ^1^
Urban	7.161 ± 0.47	6.602 ± 0.40	6.644 ± 0.44	0.000 ^2^	4.371 ± 0.27	4.003 ± 0.37	4.056 ± 0.34	0.000 ^5^
Semi-urb	7.082 ± 0.31	6.527 ± 0.35	6.333 ± 0.33	0.000 ^3^	4.378 ± 0.26	3.756 ± 0.23	3.575 ± 0.17	0.000 ^6^
Rural	7.062 ± 0.40	6.578 ± 0.40	6.360 ± 0.46	0.000 ^4^	4.444 ± 0.31	3.922 ± 0.35	3.719 ± 0.38	0.000 ^1^
*p* **	0.732	0.696	0.000 ^7^		0.056	0.001 ^8^	0.000 ^9^	

T1: first trimester; T2: second trimester, and T3: third trimester of pregnancy. * Statistical analysis between trimesters. ** Statistical analysis between socioeconomic areas.^1^ Kruskal–Wallis test. pairwise comparison: *p* = 0.000 for T1 and T2, T1 and T3, and T2 and T3. ^2^ ANOVA. Tukey test: *p* = 0.000 for T1 and T2, and for T1 and T3. ^3^ ANOVA. Tukey test: *p* = 0.000 for T1 and T2, and for T1 and T3; *p* = 0.009 for T2 and T3. ^4^ Kruskal–Wallis test. Pairwise comparison: *p* = 0.000 for T1 and T3, and for T2 and T3; *p* = 0.002 for T1 and T3. ^5^ Kruskal–Wallis test. Pairwise comparison: *p* = 0.000 for T1 and T2, and for T1 and T3. ^6^ Kruskal–Wallis test. Pairwise comparison: *p* = 0.000 for T1 and T2, and T1 and T3; *p* = 0.016 for T2 and T3. ^7^ Kruskal–Wallis test. Pairwise comparison: *p* = 0.000 between urban and rural groups; *p* = 0.010 between urban and semi-urban groups. ^8^ Kruskal–Wallis test. Pairwise comparison: *p* = 0.001 between urban and semi-urban groups; *p* = 0.005 between semi-urban and rural groups. ^9^ Kruskal–Wallis test. Pairwise comparison: *p* = 0.000 between urban and semi-urban groups; and between urban and rural groups; *p* = 0.038 between semi-urban and rural groups.

**Table 3:** Prevalence of low plasma total proteins and low albumin in the study.

	Deficit of Protein (<6.4 g/dL)			Deficit of Albumin (<3.4 g/dL)		
	T1 n (%)	T2 n (%)	T3 n (%)	T1 n (%)	T2 n (%)	T3 n (%)
Total	14 (6.51)	81 (37.67)	121 (56.28)	0	7 (3.26)	21 (9.77)
mean	6.21	6.19	6.1		3.24	3.24
(95% CI)	(6.12–6.30)	(6.16–6.22)	(6.05–6.14)		(3.17–3.32)	(3.20–3.28)
Urban	1 (2.94)	11 (32.35)	9 (26.47)	0	1 (2.94)	2 (5.88)
mean		6.14	6.13			3.25
(95% CI)		(6.03–6.25)	(5.86–6.40)			(2.61–3.89)
Semi-urban	1 (1.92)	18 (34.62)	34 (65.38)	0	1 (1.92)	5 (9.62)
mean		6.15	6.14			3.26
(95% CI)		(6.08–6.22)	(6.08–6.20)			(3.15–3.37)
Rural	12 (9.30)	52 (40.31)	78 (60.46)	0	5 (3.88)	14 (10.85)
mean	6.22	6.21	6.07		3.26	3.23
(95% CI)	(6.11–6.33)	(6.18–6.25)	(6.02–6.13)		(3.19–3.33)	(3.17–3.29)
*p* *	0.125	0.607	0.001		0.794	0.677

T1: first trimester; T2: second trimester, and T3: third trimester of pregnancy. * Statistical analysis between socioeconomic areas. Chi-square test.

**Table 4:** Levels of plasma total protein and albumin dichotomized according to the appearance of edema in pregnant women.

	Total Protein (g/dL)			Albumin (g/dL)		
	Edema +	Edema −	*p*	Edema +	Edema −	*p*
T1	7.38 (5.28–9.47)	7.08 (7.03–7.13)	0.178	4.50 (3.23–5.77)	4.42 (4.38–4.46)	0.679
T2	6.44 (6.35–6.53)	6.60 (6.54–6.66)	0.036 *	3.78 (3.71–3.85)	3.92 (3.87–3.98)	0.007 *
T3	6.29 (6.18–6.39)	6.44 (6.37–6.51)	0.008 *	3.61 (3.55–3.68)	3.79 (3.72–3.85)	0.001 *

Values are expressed as means and 95% confidence interval. Edema +: women with edema. Edema −: women without edema. T1: first trimester; T2: second trimester, and T3: third trimester of pregnancy. * Statistically significant differences.
